# Unique profile of antimicrobial peptide expression in polymorphic light eruption lesions compared to healthy skin, atopic dermatitis, and psoriasis

**DOI:** 10.1111/phpp.12355

**Published:** 2017-11-13

**Authors:** VijayKumar Patra, Gerlinde Mayer, Alexandra Gruber‐Wackernagel, Michael Horn, Serena Lembo, Peter Wolf

**Affiliations:** ^1^ Research Unit for Photodermatology Medical University of Graz Graz Austria; ^2^ Center for Medical Research Medical University of Graz Graz Austria; ^3^ Department of Dermatology Medical University of Graz Graz Austria; ^4^ Department of Medicine, Surgery, and Dentistry Scuola Medica Salernitana University of Salerno Fisciano Italy

**Keywords:** antimicrobial peptides, innate immunity, photosensitivity, polymorphic light eruption, skin microbiome

## Abstract

**Background:**

Polymorphic light eruption (PLE) has been attributed to type IV, most likely delayed‐type hypersensitivity response (adaptive immunity) but little is known on innate immunity, especially antimicrobial peptides (AMPs) in the disease. Abnormalities in AMP expression have been linked to pathological skin conditions such as atopic dermatitis (AD) and psoriasis.

**Methods:**

Antimicrobial peptide profiling was carried out in PLE skin samples (n,12) compared with that of healthy (n,13), atopic (n,6), and psoriatic skin (n,6).

**Results:**

Compared to healthy skin, we observed increased expression of psoriasin and RNAse7 (both mostly in stratum granulosum of the epidermis), HBD‐2 (in the cellular infiltrate of the dermis), and LL37 (mostly in and around blood vessels and glands) in PLE lesional skin, a similar expression profile as present in psoriatic skin and different to that of AD (with little or no expression of psoriasin, RNAse7, HBD‐2, and LL37). HBD‐3 was downregulated in PLE compared to its high expression in the epidermis and dermis of healthy skin, AD, and psoriasis.

**Conclusion:**

The unique profile of differentially expressed AMPs in PLE implies a role in the pathophysiology of the disease, possibly directly or indirectly linked to the microbiome of the skin.

## 1 INTRODUCTION

Ultraviolet radiation (UVR) results in local and systemic immunosuppression.^1^, ^2^, ^3^, ^4^ This seems, on one hand, to be a crucial factor for skin cancer development, on the other, a lack of it might favor the occurrence of the most common photodermatosis polymorphic light eruption (PLE).^5^ PLE occurs in roughly 10%‐20% of the population of the Western world, mostly among young women,^6^ and is commonly characterized by itchy skin lesions of varying morphology.^5^, ^7^, ^8^ It usually occurs during spring or early summer upon exposure to sunlight and the symptoms usually subside with repeated sunlight exposure due to continuous natural photo hardening^9^, ^10^ as the summer progresses^5^ or after medical photo hardening.^10^, ^11^ The exact etiology and pathogenesis of PLE still remain a mystery; however, resistance to UV‐induced immunosuppression and type IV delayed‐type hypersensitivity (DTH) to photo‐antigens are believed to play a key role in the disease.^5^, ^12^, ^13^ The physiological occurrence of UV‐induced immunosuppression may protect healthy subjects from symptoms of PLE by suppressing the immune reaction to newly formed (photo) antigens^12^ probably due to regulatory T cells,^14^, ^15^ whereas in PLE prone subjects, a failure of immune suppression might favor the occurrence of the skin rash of the disease.

Skin cells produce small 10‐50 amino acid residues known as antimicrobial peptides (AMPs) which have the potential to neutralize invading microorganisms.^16^ AMPs can be classified into defensins (α‐ and β‐defensins) and cathelicidin (LL37),^17^ and other AMPs such as ribonuclease 7 (RNase7), psoriasin (S100A7), and dermcidin (sweat gland derived).^18^ AMPs also take part in activating and mediating adaptive immune response.^19^, ^20^, ^21^, ^22^, ^23^ Dysregulation in AMP production has been linked to many pathological skin conditions such as psoriasis, atopic dermatitis (AD), rosacea, and others.^18^ We hypothesize that there might be an overall altered expression of AMPs as well in PLE lesions induced directly by UVR or due to possible UV‐induced damage to certain microbes or microbial elements within or on the surface of the skin and such events could contribute to the pathogenesis of the disease.^12^, ^24^ We herein investigate the expression of psoriasin, RNase7, HBD‐2, HBD‐3, and LL‐37, as these are the most commonly studied AMPs linked to many disease pathologies, using immunohistochemical stainings of lesional skin of PLE and compare it with that of healthy skin, lesional skin of AD and psoriasis patients.

## 2 MATERIALS AND METHODS

### 2.1 Samples

Formalin‐fixed paraffin‐embedded (FFPE) skin lesional biopsies of PLE of 12 patients (8 women and 4 men; median age: 60 years [range, 16‐75]; from trunk: 1; and extremities: 11), lesional skin of 6 patients with AD (1 woman and 5 men; median age: 43 [range, 6‐63]; from trunk: 3; and extremities: 3), and lesional skin of 6 patients with psoriasis (6 women; median age: 50 years [range, 31‐74]; from face/head: 1; trunk:1; and extremities: 4) as well as samples from healthy, normal looking skin of 13 individuals (6 women and 7 men; median age: 72 years [range, 47‐88]; from face/head: 8; trunk: 2; and extremities: 3) were available for the study. The healthy tissue samples were from tumor adjacent skin, obtained during surgical excision of lesions such as nevi and nonmelanoma skin cancers. The study was approved by the Ethics Committee of Medical University of Graz, Graz, Austria (18‐068 ex 06/07 and 25‐293 ex 12/13) and was performed in accordance with the guidelines of the Declaration of Helsinki Principles.

### 2.2 Immunohistochemical staining

FFPE tissue sections (3.5 μm) were deparaffinized and rehydrated for immunohistochemical staining. Slides with tissues sections were incubated for heat‐induced antigen retrieval in Dako Target Retrieval Solution Citrate pH 6.0 (Dako S2369) or Dako Target Retrieval Solution pH 9,0 (Dako S2367) for 30 minutes in a steamer. The staining was then performed manually at 4°C antibody incubation using the Dako REAL™ Detection System, Peroxidase/AEC, using monoclonal antibodies directed against: HBD2 (1:400; #ab63982, Abcam Cambridge, U.K.), HBD3 (1:100; #LS‐B86, LSbio Seattle, WA), psoriasin (1:300; #MA1‐91555, Thermo Fisher Scientific, Pittsburgh, PA), RNase7 (1:50; #ab154143, Abcam Cambridge, U.K.) and LL37 (1:50; #63982, Abcam Cambridge, U.K.). Images of stainings were acquired with a DP71 digital camera (Olympus, Vienna, Austria), attached to an Olympus BX51 microscope.

### 2.3 Quantitative analysis of AMP expression

Visual analysis was performed by counting positively stained cells in 5 randomly selected microscopic fields at a magnification of 40× using an ocular grid with area coverage of 0.25 mm^2^. For psoriasin, the number of positively stained cells were counted in the epidermis. For HBD‐2, positive cells and negative cells (HBD‐2 unstained) were counted in the dermis, and the percentage of positive cells ((number of positive cells/number of positive + negative cells)×100) was determined. For LL‐37, the number of positive blood vessels in the dermis was determined. Scoring of microscopic slides was performed in a blinded manner. Results of visual counts were averaged per patient and used for statistical analysis. For RNase7 and HBD‐3, we quantified expression using ImmunoRatio plugin^25^ in ImageJ software with the images acquired with the Olympus DP71 digital camera. The percentage of the DAB‐stained nuclear area over the total nuclear area in epidermis and dermis was calculated, and percentages were subjected to statistical analysis.

### 2.4 Statistical analysis

Statistical analysis was performed using GraphPad Prism 6. For immunohistochemistry score comparison, unpaired nonparametric, Kruskal‐Wallis test was used; each *P*‐value was adjusted to account for multiple comparisons. A *P*‐value smaller than .05 has been set as statistically significant.

## 3 RESULTS

**Figure 1:**
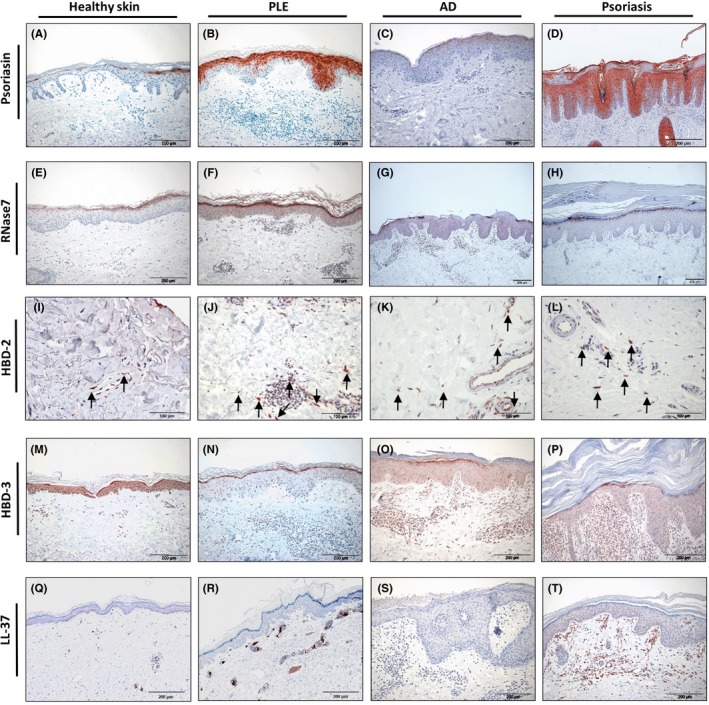
Immunohistochemical staining reveals altered antimicrobial peptide expression in polymorphic light eruption. Representative images of immunohistochemically stained tissue sections (counterstained with hematoxylin). Polymorphic light eruption (PLE), atopic dermatitis (AD), and psoriasis samples were stained for psoriasin (A‐D), RNase7 (E‐H), HBD‐2 (I‐L), HBD‐3 (M‐P), and LL37 (Q‐T). Healthy, normal‐appearing human skin was used as a control. PLE showed increased expression of Psoriasin (B) and RNAse7 (F) both mostly in the stratum granulosum of the epidermis; HBD‐2 was mostly expressed in the cellular infiltrate in the dermis (J) and LL37 in and around blood vessels and glands (R), whereas HBD‐3 (N) was decreased in epidermis and dermis. A similar expression profile is observed in lesional psoriatic skin, different to that of lesional skin of AD (with little or no expression of psoriasin, RNAse7, HBD‐2 and LL37 and upregulation of HBD‐3). Original magnification of psoriasin, RNase7, HBD‐3, and LL37: ×200 and HBD‐2: ×100

**Figure 2:**
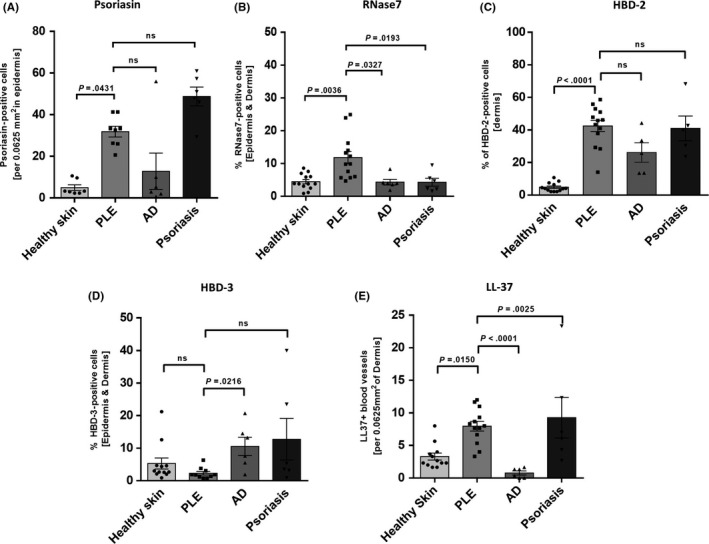
Quantitative analysis of the immunohistochemical stainings of antimicrobial peptide expression. (A) In polymorphic light eruption (PLE), the number of psoriasin‐positive cells in the epidermis was significantly higher than in healthy skin but slightly lower compared to that in psoriasis. (B) Expression of RNase7 in PLE was significantly higher than compared to that of healthy skin, atopic dermatitis (AD) or psoriasis. (C) HBD‐2 was significantly increased in PLE and psoriasis and to a lower degree increased in AD compared to healthy skin. (D) The expression of HBD‐3 was (significantly) decreased in PLE compared to AD, psoriasis, and healthy skin. (E) Similar to psoriasis, PLE showed significantly increased expression of LL37 compared to healthy skin and AD. Data presented as mean with SEM

Psoriasin was the most highly expressed by keratinocytes in PLE and psoriasis (Figure 1B,D); it was expressed in the nucleus and cytoplasm (of the) stratum granulosum and stratum spinosum, whereas the basal layers and stratum corneum showed no expression in PLE, but little expression in psoriasis. In contrast, AD (Figure 1C) showed very little expression of psoriasin (only in stratum granulosum of the epidermis). In healthy skin (Figure 1A), expression of psoriasin was found in stratum granulosum but was less intense and patchy compared to PLE or psoriasis. Overall, the expression of psoriasin in PLE was statistically significantly higher compared to healthy skin (Figure 2A).

Ribonuclease 7 was expressed in PLE (Figure 1F), mainly in keratinocytes of the stratum granulosum and in the stratum corneum and less intensively in the basal layers of the epidermis. Healthy skin and AD (Figure 1E,G) showed very low expression of RNase7 in stratum corneum and stratum granulosum. Psoriasis (Figure 1H) showed a high level of expression of RNase7 in stratum corneum, but subtle expression in other layers of the epidermis. Altogether the expression of RNase7 in PLE was significantly higher than in healthy skin or lesional skin of AD and psoriasis (Figure 2B).

HBD‐2 was expressed in infiltrating cells in the dermis in PLE, AD, and psoriasis (Figure 1J,K,L), whereas healthy skin (Figure 1I) showed no expression or very low expression of it. PLE showed significantly higher numbers of HBD‐2‐positive cells in the dermis compared to healthy skin (Figure 2C).

HBD‐3 expression was only modestly expressed in the stratum granulosum but not at all in the other layers of the epidermis in PLE (Figure 1N). In contrast, healthy skin (Figure 1M), AD (Figure 1O) and psoriasis (Figure 1P) showed higher expression of HBD‐3 in epidermal layers as well as infiltrating cells in the dermis. Statistical significance was reached when PLE was compared to AD (Figure 2D).

LL‐37 was profoundly expressed in and around blood vessels and glands in PLE and psoriasis (Figure 1R,T). Healthy skin or AD (Figure 1Q,S) showed no expression or very low expression of LL37. Overall, the increased expression of LL‐37 in PLE and psoriasis was significant compared to healthy skin or AD (Figure 2E).

Statistical correlation analysis revealed the expression of AMPs was independent of age, except for psoriasin in PLE (*r* = .7012, *P* = .0095). Moreover, there was no overall statistical correlation between sex and expression of any of the AMPs of this study.

## 4 DISCUSSION

AMPs are produced in the skin during inflammation and/or in cases of infection. It has been demonstrated that UVR can induce production of keratinocyte‐derived AMPs both in vitro and in vivo in humans and rodents.^26^, ^27^, ^28^, ^29^, ^30^ Previous work on normal human keratinocytes revealed a dose‐dependent increase in human β‐defensin‐2, β‐defensin‐3, RNase7, and psoriasin (S100A7) after UVB radiation. Note should be taken that interindividual variations are observed in expression of AMPs by UVR.^27^, ^29^, ^31^ We now report a unique profile of protein expression of psoriasin, RNase7, HBD‐2, HBD‐3, and LL‐37 in PLE occurring after natural sunlight exposure.

Psoriasin is reported to be expressed in inflamed skin and can exert a chemotactic influence on inflammatory cells.^32^ In our study, psoriasin was highly expressed in PLE lesions and psoriatic skin, compared to healthy skin or AD (for the latter two diseases consistent with previous reports^33^, ^34^, ^35^, ^36^). However, we did not detect psoriasin expression in AD; indeed, the data on it in AD are controversial, possibly due to differences in analytical methodologies employed.^37^ IL‐1α, IL‐1β, IL‐19, IL‐36, IL‐22, IL‐17, TNF‐α, calcium, flagellin, and UV enhance psoriasin expression.^38^, ^39^, ^40^, ^41^ In PLE, IL‐1β levels have been found to be elevated,^42^ and this could induce high psoriasin expression in the skin. Psoriasin displays a strong, selective antimicrobial activity against *Escherichia coli* strains,^40^, ^43^ and this could theoretically hint toward the involvement of *E. coli* or its components in PLE lesions which is yet to be investigated.^12^

Ribonuclease 7 is produced vastly by keratinocytes. It shows a strong bactericidal activity against a broad‐spectrum of Gram‐negative and Gram‐positive bacteria.^44^, ^45^ We found that RNase7 was more strongly expressed in lesional PLE skin than in healthy skin and AD or psoriatic skin. RNase7 is also constitutively expressed in healthy skin,^46^, ^47^, ^48^ consistent with our findings, indicating that higher expression in PLE lesions could possibly be further aggravated by bacteria. Other constitutive inducers of RNase7 expression include IL‐17A and IFN‐γ (via STAT3).^49^ A trend for elevated serum IL‐17 levels in PLE has been previously reported,^42^ suggesting that higher levels of IL‐17 could induce production of RNase7. Moreover, UVR is capable of inducing RNase7 expression.^27^ Data on RNase7 expression in AD as well as in psoriatic skin are controversial.^36^, ^37^, ^50^, ^51^, ^52^ However, we found the expression of RNase7 to be relatively low in lesional skin of AD and psoriasis patients. Notably, besides antibacterial activity RNase7 shows also immunomodulatory functions on Th2 cells and cytokine production.^53^

Human β‐defensin 2 was first isolated from extracts obtained from psoriatic skin.^54^ It is known to be upregulated in the skin by LPS, TNF‐α, IL‐1β, IL‐1α and bacterial infections^55^ and also 1,25‐dihydroxyvitamin D_3._ 56 We found HBD‐2 to be highly expressed in PLE, especially in infiltrating cells in the dermis. In this regard, it is already known in PLE that there is a lack of neutrophils and TNF‐α,^57^ but an increase in IL‐1β^58^ production which could induce the expression of HBD‐2.^42^, ^59^ In ex vivo experimental setting, the proinflammatory mediators TNF‐α and IL‐17 but not UVR stimulated the expression of HBD‐2.^60^ However, in vivo data showed more heterogeneous expression for HBD‐2 by UVR.^27^ The presence of LPS and other microbial products in PLE remains to be determined. Lande et al^61^ found that HBD‐2 and HBD‐3 have strong ability to break the tolerance to human DNA and form complexes with nucleic acids and trigger an innate immune response. It is known that the UVB waveband of sunlight can modify nucleic acids which could then become potential antigens,^5^, ^12^, ^62^ provoking an immune reaction as seen in PLE, linked to higher expression of HBD‐2 possibly exaggerating inflammation.

HBD‐3 is expressed by the same cells as HBD‐2 in the skin. We found that the expression of HBD‐3 in PLE lesions was considerably lower than in healthy skin, and lesional skin of AD and psoriasis patients. The inducers for upregulating HBD‐3 are similar to those of HBD‐2. Among other β‐defensins, HBD‐3 is the only AMP which is regulated by insulin‐like growth factors such as (IGF‐1) and transforming growth factor (TGF‐α) and microbial stimuli (such as LPS, peptidoglycan, or SpeB [a virulence factor from *Streptococcus pyogenes*]) to keratinocytes in vitro.^63^ Although UVR is known to induce expression of HBD‐3, our observations of impaired expression in PLE lesions might imply its functional role in disease pathogenesis as HBD‐3 is known to be involved with keratinocyte proliferation and migration by activating EGFR, STAT1, and STAT3.^21^ HBD‐3 is known to be more potent in killing microbes than other defensins.^64^ The lower or reduced expression of HBD‐3 in PLE lesions in our study and a previous report^26^ could suggest a differential microbial landscape in PLE patients that fails to induce HBD‐3, similarly as observed in AD where lower HBD‐3 expression corresponds to higher *Staphylococcus aureus* colonization.^65^, ^66^

LL‐37 is the only cathelicidin peptide detected in humans and produced in much vast quantity in psoriasis and is known to play a major role in the inflammatory cascade driving psoriatic disease.^67^, ^68^, ^69^, ^70^ Herein and in a previous report,^71^ we observed significant expression of this peptide around blood vessels and glands in the dermis of the PLE lesions. Our observation of increased LL‐37 expression in lesional skin of psoriasis and decreased expression in AD patients is consistent with previous findings.^72^, ^73^ LL‐37 is induced by UVB,^29^ 1,25‐dihydroxyvitamin D_3,_ 56 components of bacterial infections and stress, cytokines such as IFN‐γ, TNF‐α, IL‐6, and activated TLRs.^74^ Rather low systemic levels of vitamin D have been reported in PLE and other photodermatoses^75^; hence, LL‐37 similar as Tregs could be induced in PLE by other, nonvitamin D‐dependent factors.^76^ Previous research indicates that LL‐37 is a vital mediator in activating pDCs by forming aggregates with self‐nucleic acids.^68^, ^69^, ^70^ However, in PLE patients a complete absence of pDCs has been reported,^77^ suggesting that the high expression of LL‐37 might be involved in other inflammatory processes. In psoriasis patients, LL‐37 has been recognized as an autoantigen that stimulates circulating T cells and contributes to the autoimmunity in these patients.^78^ As a similar autoimmune environment may obviously exist in PLE patients, it is likely that there are increased LL‐37‐specific T cells. On the other hand, LL‐37 shows broad antimicrobial activity to various microbes and the high expression of this peptide could be directly involved in the antimicrobial activity in PLE patients.

The major limitations of this study were the limited sample size and the imperfect matching in age and sex of the different patient groups. However, our analysis showed that the expression of AMPs (except for psoriasin that was age‐related in PLE) was neither age‐ nor sex‐dependent in the sample sets of the study. Indeed, age‐matched sample (data not shown) analysis revealed a similar picture than the overall analysis shown in Figure 2. Furthermore, previous work has shown that psoriasin, RNase7, and HBD‐3 was not only upregulated in UVR‐provoked lesional PLE skin but also to a weaker extent in UVR‐exposed skin of PLE patients without eruption. However, as our study lacks normal skin from PLE patients, we cannot conclude on the magnitude of AMP expression by UVR without PLE eruption and if UVR increases AMP expression in PLE lesions above levels of nonlesional skin of PLE patients not mounting PLE after UV exposure. Hence, the results of this study must be interpreted in the light of the study's limitations. Previous research has shown consistently with our results a certain extent of altered expression levels of AMPs (psoriasin, RNase7, HBD‐2, HBD‐3) in a limited number of PLE lesions photo‐provoked by artificial UV‐A radiation.^26^ Our findings of the unique expression pattern of AMPs, including LL‐37 (that at least hypothetically could be a potential driver in PLE) provide further understanding of the pathogenesis of the disease and could help unraveling a complex network between AMPs, microbiome, and immune system. That said, the potential case of altered microbial landscape in PLE patients is yet to be investigated, but the altered expression of various AMPs among different skin conditions in our study strongly implies that either microbial elements or microbes themselves may be involved in the pathogenesis of PLE. Indeed, these microbial elements could be the source of the yet undetected antigens formed in PLE patients after exposure to UVR.^12^

## AUTHORS' CONTRIBUTIONS

VP and PW had full access to all of the data in the study and took responsibility for the integrity of the data and the accuracy of the data analysis. VP and PW were involved in study concept and design. VP and PW were involved in acquisition, analysis, and interpretation of data. VP and PW were involved in drafting of the manuscript. All authors critically revised the manuscript for important intellectual content. VP and PW statistically analyzed the data of the manuscript. Wolf obtained the funding for the research presented in the manuscript. GM, AG‐W, MH, and SL were involved in administrative, technical, or material support. PW supervised the study.

## CONFLICT OF INTEREST

None reported.
